# Eco-Epidemiology and Pathological Impact of *Capillaria contorta* (Capillariidae) in Semi-Captive Barbary Partridges (*Alectoris barbara*) in Algeria

**DOI:** 10.3390/ani16121781

**Published:** 2026-06-09

**Authors:** Djeloud Messaouda, Saadi-Idouhar Habiba, Belhadj-Kebbi Melaaz, Srisupaph Poonlaphdecha, Dahmani Abla, Alexis Ribas

**Affiliations:** 1 Laboratory of Applied Zoology and Animal Ecophysiology, Faculty of Nature and Life Sciences, University of Bejaia, Bejaia 06000, Algeria; messaouda.djeloud@univ-bejaia.dz (D.M.); kmelaaz@yahoo.fr (B.-K.M.); 2 Laboratory of Research Management of Local Animal Resources (GRAL), Higher National Veterinary School, Abbes Street, Oued-Smar, Algiers 16000, Algeria; h.saadi@ensv.dz; 3 Parasitology Section, Department of Biology, Healthcare, and Environment, Faculty of Pharmacy and Food Science, University of Barcelona, 08028 Barcelona, Spain; spoonlaphdecha@ub.edu; 4 Institute of Research in Biodiversity (IRBio), University of Barcelona, 08028 Barcelona, Spain; 5 Zeralda Hunting Center, Zeralda, Algiers 16000, Algeria; dahmaniabla788@gmail.com

**Keywords:** Barbary partridge (*Alectoris barbara*), *Capillaria*, co-infection, histopathology, parasite prevalence, Algeria, semi-captive breeding

## Abstract

The Barbary partridge (*Alectoris barbara*), an endemic game bird in Algeria, has not been systematically investigated for parasitic infections despite its ecological, economic, and conservation importance. This study provides the first eco-epidemiological and histopathological assessment of *Capillaria contorta* infection in birds from a semi-captive breeding center in Zeralda, Algeria. Between 2021 and 2024, 1085 pooled fecal samples and 138 postmortem examinations were analyzed using standard parasitological and histopathological methods. *C. contorta* showed an aviary-level overall prevalence of 32.94% and was involved in 42.02% of parasite-related mortalities, underscoring its marked pathological significance. Aviary-level prevalence varied significantly across seasons and years (*p* < 0.05), with higher levels recorded during environmentally favorable periods. Mixed infections with helminths, protozoa, cestodes, and ectoparasites were recorded for the first time in this host species at the aviary level, highlighting complex parasite interactions. Morphological characteristics were consistent with *C. contorta*. Gross examination revealed marked thickening of the esophagus and crop. Histologically, the adult nematodes were embedded within hyperplastic epithelium, with characteristic bipolar-plugged eggs and associated epithelial tunnels, accompanied by chronic inflammation and granulomatous reactions. These findings demonstrate the clinical relevance of *C. contorta* and emphasize the need for strengthened surveillance and control programs to reduce infection pressure and prevent potential spillover to wild populations.

## Simple Summary

This study presents the first eco-epidemiological and histopathological investigation of *Capillaria contorta* infection in the Barbary partridge (*Alectoris barbara*) in Algeria, a species of importance for hunting and conservation programs. Over a four-year period (2021–2024), 1085 fecal samples and 138 necropsies were analyzed from a semi-captive breeding population at the Zeralda Hunting Center. Overall fecal positivity for parasites reached 77.8%, indicating widespread exposure, with *C. contorta* detected at a prevalence of 32.94%. Despite its classification as a satellite species, *C. contorta* was implicated in 42.02% of parasite-related mortalities, highlighting its significant pathological impact. Infections occurred exclusively as mixed infections, most frequently in association with *Syngamus trachea*, *Strongyloides* sp., and *Eimeria* spp., suggesting that co-infections may increase disease severity. Significant annual and seasonal variations were observed, with peak prevalence in spring, likely influenced by environmental conditions affecting parasite survival and transmission. Morphological and histopathological findings were consistent with *C. contorta*, revealing severe epithelial hyperplasia, chronic inflammation, and fibrosis in the crop and esophagus. These findings demonstrate the clinical and epidemiological importance of capillariosis and highlight the need for improved parasite monitoring and management strategies to protect animal health and reduce the risk of transmission to wild populations.

## 1. Introduction

The Barbary partridge (*Alectoris barbara*) is a North African game bird belonging to the family Phasianidae and represents an emblematic component of Mediterranean ecosystems [1]. In Algeria, it occupies diverse ecological zones ranging from northern pre-forest habitats to southern pre-Saharan regions, with population densities influenced by habitat structure and environmental conditions [2]. Outside mainland North Africa, established populations occur in Gibraltar (south of the Iberian Peninsula), the Canary Islands (Atlantic Ocean), and Sardinia Island (Mediterranean Sea), where the species was likely introduced historically [3,4]. Its population dynamics are shaped by multiple factors, including rainfall variability, predation pressure, and habitat quality [5]. Although currently classified as “Least Concern” on the IUCN Red List [6], the species remains protected under several international and national conservation frameworks.

Despite its ecological, cultural, and economic importance in Algeria—particularly for hunting and game management—knowledge of the parasitic fauna of *A. barbara* remains limited. Semi-captive breeding programs have recently been developed to reinforce wild populations and support hunting activities, notably at the Zeralda Hunting Center, the principal breeding facility for this species in Algeria. However, parasitic diseases, especially helminth infections, constitute a major health constraint in both captive and wild populations. High stocking densities, favorable microclimatic conditions, and the presence of intermediate hosts facilitate parasite transmission [7]. Consequently, polyparasitism is frequent and may enhance pathogenic effects, increasing morbidity and mortality.

Among helminths infecting galliform birds, nematodes of the genus *Capillaria* (Capillariidae) are widely distributed and commonly reported worldwide [7,8]. These slender nematodes parasitize various organs depending on the species, including the upper digestive tract (crop and esophagus), intestines, respiratory tract, and liver. Infections are typically chronic and may impair host condition, particularly under high parasite burdens or concurrent infections.

Species of *Capillaria* have been reported in several species of the genera *Alectoris* and *Perdix*, including *A. rufa*, *A. chukar*, *A. graeca*, and *P. perdix* in Europe and Asia [9,10,11,12,13,14,15,16,17,18]. However, no dedicated eco-epidemiological and histopathological study has specifically investigated *Capillaria* infections in *A. barbara*. The life cycle of species of *Capillaria* may be direct, through ingestion of embryonated eggs, or indirect, involving intermediate hosts, suggesting that seasonal and ecological factors likely influence transmission dynamics [19]. Nevertheless, temporal patterns remain poorly documented in partridge species, particularly under North African conditions.

Among the most pathogenic avian species are *Capillaria annulata* and *C. contorta* (syn. *Eucoleus annulatus* and *Eucoleus contortus*), which parasitize the crop and esophagus [7,20]. Although historically distinguished, these taxa are now generally regarded as synonymous, with *C. contorta* recognized as the valid species [20]. In poultry, infections may cause severe mucosal damage, chronic inflammation, crop dilation, progressive emaciation, and occasionally death [7].

Previous investigations in *A. barbara* have been limited to coprological surveys or gastrointestinal tract dissection, including one preliminary study in Algeria [21] and two isolated reports, one from Sardinia Island (Italy) and the other from Tenerife Island (Canary Islands, Spain) [17,22]. To address this gap, the present study provides the first comprehensive eco-epidemiological and histopathological investigation of *C. contorta* in Barbary partridges in Algeria. Over a four-year period, we evaluated aviary-level prevalence, co-infection patterns, and seasonal dynamics, and we present the first detailed histopathological characterization of capillariosis in *A. barbara*, focusing on lesion localization and tissue responses in the crop and esophagus.

## 2. Materials and Methods

### 2.1. Study Site

The study was conducted at the Zeralda Hunting Center (36°42′03.7″ N, 2°51′40.5″ E), located in northern Algeria, approximately 30 km west of Algiers and 2 km from the Mediterranean coast. Established in 1970, the center is a public scientific and technological research institution dedicated to the production, management, and conservation of game species, including the Barbary partridge (*A. barbara*), Chukar partridge (*A. chukar*), common pheasant (*Phasianus colchicus*), and Barbary deer (*Cervus elaphus barbarus*). It is currently the only facility in Algeria equipped with a specialized breeding unit for *A. barbara*.

The region is characterized by a sub-humid Mediterranean climate with mild, wet winters and warm, dry summers. Rearing aviaries are surrounded by dense natural vegetation (*Olea europaea*, *Asphodelus microcarpus*, *Urtica dioica*, *Pistacia lentiscus*, *Triticum durum*, *Hordeum vulgare*, *Vicia sativa*, *Lotus corniculatus*), creating environmental conditions conducive to semi-captive management and potential exposure to parasite reservoirs.

The target species, *A. barbara*, had an estimated annual adult population ranging from 800 to 1035 individuals. Birds were maintained in isolated outdoor enclosures containing natural vegetation and artificial shelters to reduce climatic stress. Field investigations were conducted from September 2021 to June 2024.

### 2.2. Semi-Captive Management Conditions

Partridges were maintained under semi-captive conditions in conservation aviaries (~750 m^2^) from spring to mid-winter. Stocking density averaged 2.3 birds/m^2^, with a mean population size of approximately 780 individuals. Breeding stock was introduced in February; females were placed first, followed by the gradual introduction of males in small groups. During the reproductive season, two laying units were used, consisting of 26 cages (approximately 30 birds per cage; sex ratio 1:1) and two larger aviaries housing the remaining breeders.

### 2.3. Sampling Design

Sampling was conducted over four consecutive years (September 2021–June 2024) to evaluate parasite prevalence and its seasonal and interannual variation. A mixed longitudinal design was employed: core aviaries were sampled repeatedly throughout the study period, while new aviaries were progressively added as the breeding population expanded. Sampling frequency was adjusted according to presumed transmission risk: weekly during periods of lower transmission (summer and autumn) and biweekly during higher-risk periods (winter and spring). Carcasses were collected opportunistically throughout the study, particularly during suspected parasitic outbreaks.

At each sampling session, three to four pooled fresh fecal samples were collected per aviary, yielding a total of 1085 samples. Samples were collected early in the morning, placed in labeled sterile containers (species, enclosure, date), stored in cooled containers, and transported promptly to the Parasitology Laboratory of the Higher National Veterinary School of El-Harrach for analysis.

### 2.4. Coprological Examination

Fecal samples were first examined macroscopically to assess consistency and detect visible parasitic elements. Coprological analysis was performed using a flotation technique with saturated sodium chloride solution (specific gravity 1.20) [23]. Approximately 5 g of feces was homogenized in flotation solution, filtered, and transferred to test tubes to form a convex meniscus. A coverslip was placed on top and left for 15–20 min to allow for the flotation of parasitic stages. The coverslip was then mounted on a slide and examined under light microscopy at ×100 and ×400 magnifications. Parasites were identified based on morphological criteria using standard taxonomic keys [24,25,26,27].

For *Eimeria* spp., oocysts were concentrated by flotation and allowed to sporulate in potassium dichromate solution at room temperature for 2–5 days until complete sporulation. Sporulated oocysts were examined at ×400–×1000 magnification and identified at the genus level according to established morphological criteria [27].

### 2.5. Postmortem Examination

A total of 138 carcasses were examined immediately after collection. Each carcass was individually placed in a numbered, sealed white plastic bag labeled with the date, location, and species name to prevent ectoparasite escape. Ectoparasites were collected by allowing them to leave the carcass naturally onto the bag. The contents were then examined under a magnifying glass (Leica, Wetzlar, Germany) and preserved in 70% alcohol. For identification, specimens were cleared in potassium hydroxide (KOH) for at least 24 h, rinsed with distilled water, and mounted for microscopic examination. Complete necropsies were performed following standard avian pathology procedures. The entire digestive tract was removed and examined macroscopically for adult parasites and associated lesions. Each segment (trachea, esophagus, crop, proventriculus, gizzard, duodenum, jejunum, ileum, ceca, rectum, and cloaca) was examined first with the naked eye and subsequently under a stereomicroscope (Leica, Wetzlar, Germany) (×20–×40 magnification). The segments were then separated and inspected individually. Luminal contents were collected and analyzed using the same sodium chloride (NaCl) flotation method (specific gravity 1.20).

### 2.6. Histopathological Analysis

Fresh tissue samples from the esophagus, crop, proventriculus, gizzard, duodenum, jejunum, ileum, and ceca were collected during necropsy. Particular attention was given to the esophageal and crop mucosa for the detection of adult *Capillaria* and their eggs.

Tissues were fixed in 10% buffered formaldehyde solution for 24 h, dehydrated in graded ethanol (70%, 80%, 90%, 95%, and 100%), cleared in toluene, and embedded in paraffin. Sections (5 μm) were cut using a microtome (Leica, Wetzlar, Germany) at the Anatomopathology Laboratory, National Veterinary School of Algiers, Algeria and stained with hematoxylin and eosin according to standard protocols [28]. Slides were examined using a Leica light microscope (Leica Microsystems, Wetzlar, Germany) equipped with a digital imaging system. Lesions were evaluated for epithelial alterations, inflammatory infiltrates, granuloma formation, and the location of the parasite.

Morphometric measurements of adult worms (body diameter) and eggs (length and width) were obtained using ImageFocusAlpha software version v1.3.7.31026 (260322) (University of Barcelona, Barcelona, Spain) and AmScope software version 3.7.9432.20170726 (AmScope Imaging, Irvine, CA, USA) (ENSV, El-Harrach, Algiers).

### 2.7. Statistical Analysis

Prevalence was calculated as the proportion of positive samples among those examined, with 95% confidence intervals. The overall, annual (2021–2024), and seasonal aviary-level prevalence rates of *Capillaria* were determined. Differences in prevalence between seasons and years were assessed using the chi-square (χ^2^) test, with significance set at *p* < 0.05.

Parasite species were categorized according to the prevalence-based classification of Bush and Holmes [29] as central (≥66.6%), secondary (33.3–66.6%), or satellite (≤33.3%). Morphometric data for *C. contorta* eggs (length and width) were summarized descriptively to assess variability. All statistical analyses were performed using SPSS version 27 (IBM Corp., Armonk, NY, USA).

### 2.8. Ethical Statement and Biosecurity Protocols

This study examined naturally deceased birds from the Cynegetic Center of Zeralda. No live animals were captured or sacrificed. Pooled fecal samples per aviary were collected non-invasively during early morning routine monitoring, in cooperation with the Centre’s veterinarian. Necropsy examination was conducted at the Algiers School of Veterinary Medicine with veterinary professionals, ensuring ethical standards and professional gross pathological assessment. Sampling was suspended if disease outbreaks or abnormally high mortality occurred. All specimens were handled ethically and disposed of appropriately. Parasitological and histological examinations followed standard biosafety protocols.

## 3. Results

Coprological examination revealed *C. contorta* in association with other parasitic taxa belonging to four major groups: coccidia, nematodes, cestodes, and ectoparasites. Infections with *C. contorta* occurred exclusively in mixed-species assemblages; no single-species infections were detected.

Overall aviary-level fecal positivity for at least one parasitic taxon was 77.8% (844/1085). The aviary-level prevalence of *C. contorta*, calculated on the total number of positive samples, was 32.94% (278/844), classifying it as a satellite species according to prevalence-based criteria. Among parasite-related mortalities (58/138 necropsied birds), *C. contorta* was detected in 42.02% of cases within co-infection contexts, underscoring its clinical relevance within the parasite community of *A. barbara*.

Double co-infections involving *C. contorta* represented 2.52% of cases and were most commonly associated with *Syngamus trachea*, *Eimeria* spp., and mites. Triple infections accounted for 8.99%, whereas quadruple-species associations were most frequent (19.06%). In complex co-infections (≥3 species; *n* = 58), the parasites most frequently associated with *C. contorta* were *Eimeria* spp. (50.0%), *Strongyloides* sp. (48.28%), and *S. trachea* (46.5%) (Table 1). Details on co-infections are provided in Table A1.

### 3.1. Temporal Variation in Capillaria contorta Prevalence

Analysis of temporal trends revealed significant annual and seasonal variations in aviary-level *C. contorta* prevalence between 2021 and 2024. Annual prevalence decreased significantly over the study period (χ^2^ = 18.82, *p* = 0.0003), declining from 39.36% in 2021 to 19.50% in 2024 (Table 1).

Seasonal variation, based on pooled data across the four study years, was even more pronounced (χ^2^ = 93.19, *p* < 0.000001). The highest prevalence was recorded in spring (50.72%; 95% CI: 45.46–55.98), followed sequentially by winter, autumn, and summer (Table 2). Confidence intervals were narrow and showed minimal overlap between seasons with markedly different prevalence values, supporting the robustness and biological relevance of these differences.

### 3.2. Gross Pathological Findings

**Figure 1:**
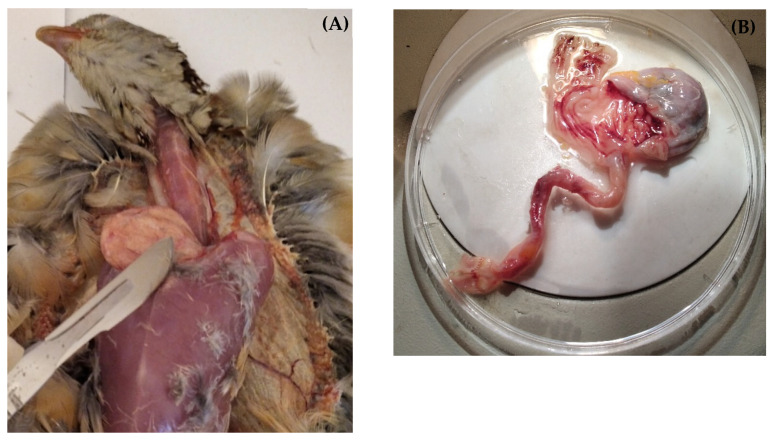
Gross pathological findings in *A. barbara* infected with *Capillaria contorta*: (**A**) emaciated carcass showing marked pectoral muscle atrophy and a distended, pendulous crop; (**B**) the esophagus and crop exhibiting mucosal congestion, thickening, vascular dilation, and intraluminal hemorrhagic exudate.

At necropsy, *C. contorta* infection was confirmed in 58 of 138 birds (42.02%). Affected individuals exhibited poor body condition, severe emaciation, and marked atrophy of the pectoral musculature (Figure 1A). The gastrointestinal tract was generally devoid of ingesta.

*Capillaria contorta* was detected exclusively in the crop and esophagus. Other examined tissues (proventriculus, gizzard, duodenum, jejunum, ileum, and ceca) did not contain parasites and showed only non-specific degenerative changes.

Pronounced deformation and thickening of the esophagus and crop were observed. On opening, the mucosa appeared congested, edematous, and thickened, with dilated vessels and linear hemorrhagic streaks. The luminal content consisted of tenacious mucus admixed with blood, without residual feed material (Figure 1B). Adult nematodes were not visible macroscopically. Detection required examination under a stereomicroscope (a binocular microscope ×40 and ×100).

### 3.3. Morphological Characteristics of Capillaria contorta

**Figure 2:**
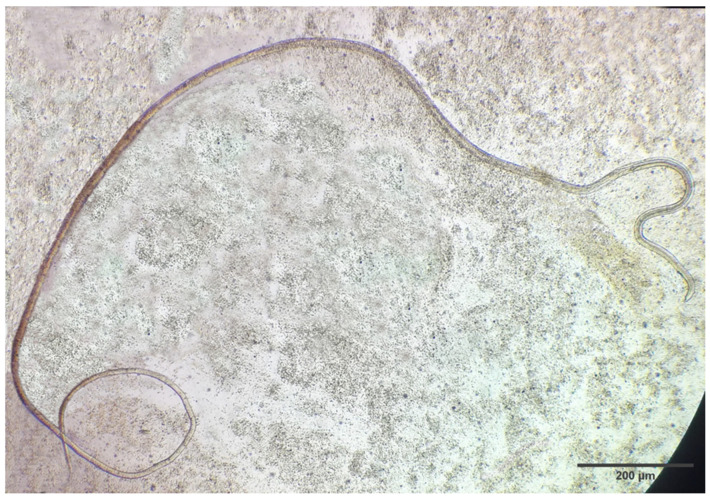
Male *Capillaria contorta* recovered from the crop mucosa, showing a slender body with a distinct esophagus and well-defined anterior and posterior extremities ×40.

A male specimen embedded within the crop mucosa was recovered (Figure 2). The worm displayed the typical filiform morphology of the genus, with a slender, translucent body measuring approximately 13 mm in length and 57–60 µm in width. The esophagus was elongated and distinct. The posterior extremity contained a single smooth spicule without spines. The absence of a cuticular swelling posterior to the cephalic region supports identification consistent with *C. contorta* rather than *C. annulata*.

### 3.4. Morphology of C. contorta Eggs

Eggs were barrel-shaped with symmetrical bipolar plugs and were embryonated at the time of detection. Egg dimensions ranged from 47–61.25 µm in length and 24–27.94 µm in width (*n* = 274). Polar plugs measured 2.91–5.14 µm in height and 5.63–10.53 µm in width. The shell was smooth, yellow-brown, and approximately 1.79 µm thick.

**Figure 3:**
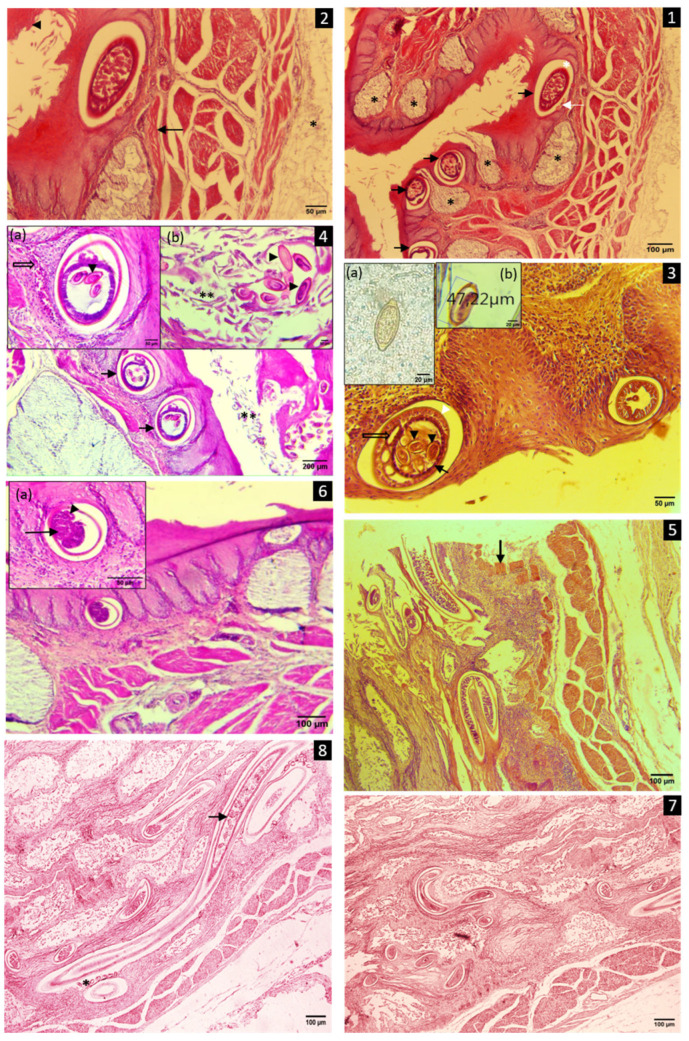
Histopathological lesions associated with *Capillaria contorta* infection in the crop and esophagus of *A. barbara* (H&E stain): (**1**) Keratinized stratified squamous epithelium with submucosal inflammatory infiltration and glandular necrosis (black asterisks); intraepithelial parasites (black arrows) with eggs (white arrow) in the interstitial space (white asterisk) ×400. (**2**) Marked epithelial hyperplasia with intraepithelial adult worms; fibrinous edema (asterisk); basal layer (arrowhead), lamina propria (black arrow) ×400. (**3**) Submucosal immune cell infiltration; detail of adult worm showing cuticle (white arrowhead), uterine wall (arrow), embryonated bipolar-plugged eggs (black arrowheads), and esophagus (empty arrow) ×400. (**3a**) Typical barrel-shaped egg with bipolar plugs observed in feces ×400. (**3b**) Embryonated egg with morphometric measurement ×400. (**4**) Cross-section of a gravid female within the crop epithelium (black arrow). ×100. (**4a**,**4b**) Higher magnification showing intrauterine eggs (arrowhead) eggs dispersed in surrounding connective tissue (empty arrow). General alteration of the epithelium is manifested by extensive cellular desquamation (**) ×400. (**5**) Chronic esophageal inflammation with basement membrane thickening (arrow) and diffuse inflammatory infiltration ×400. (**6**) Cross-sections of male worms within epithelial tunnels ×100. (**6a**) Male reproductive structures with germ cells and mature spermatozoa (arrow); intestine visible (arrowhead) ×400. (**7**,**8**) Multiplanar sections of adult worms within the esophageal epithelium; elongated reproductive tract containing characteristic eggs (arrow); extraparasitic eggs in surrounding tissue (*) ×400.

Histologically, eggs at various developmental stages were observed within epithelial tunnels excavated in the hyperplastic stratified squamous epithelium of the crop and esophagus, as well as free within the mucosa (Figure 3).

### 3.5. Histopathological Findings

Histological sections from crop and esophageal tissues contained *C. contorta* as the sole parasitic element; parasites detected in other anatomical regions during necropsy dissection (*Syngamus trachea*, *Eimeria* spp., *Ascaridia galli*, *Heterakis gallinarum*, and *Acarina* spp.) were not present in histological sections.

Severe lesions were consistently observed in the crop and esophagus of infected birds. Marked hyperplasia of the stratified squamous epithelium was associated with extensive intraepithelial localization of adult worms. The lamina propria and submucosa exhibited dense chronic inflammatory infiltrates composed predominantly of lymphocytes, plasma cells, and numerous eosinophils. Submucosal fibrosis and collagen deposition indicated chronicity.

Heavy parasite burdens were associated with degeneration of the muscular layer and focal necrosis of mucous glands. Numerous transverse and longitudinal sections of adult worms were embedded within epithelial tunnels (Figure 3). Parasites were irregularly distributed, with cross-sectional diameters ranging from 98.7 to 322.57 µm (*n* = 91). Internal structures, including smooth cuticle, musculature, bacillary bands, intestines, and female reproductive organs, were clearly identifiable. The mean uterine diameter in females was approximately 136 µm (*n* = 60).

## 4. Discussion

The present study provides the first comprehensive eco-epidemiological and histopathological characterization of *C. contorta* infection in the Barbary partridge (*A. barbara*). Although *Capillaria* infections have been reported in several Phasianidae species within the Mediterranean basin, including the Cantabrian capercaillie (*Tetrao urogallus cantabricus*) and the common pheasant (*P. colchicus*) in Spain [19,30], no previous investigation has integrated epidemiological, pathological, and temporal analyses in *A. barbara*. Earlier experimental and field studies demonstrated that infestation of the crop and esophagus by *C. annulata* (currently recognized as *C. contorta*) causes severe pathological consequences in galliform birds, including growth retardation, decreased egg production, reduced fertility, and increased mortality [31].

According to the ecological framework of Bush and Holmes [29], *C. contorta* was classified here as a satellite species (prevalence 32.94%). Satellite species typically exhibit aggregated distributions and strong dependence on specific ecological conditions. In this case, transmission likely depends on the availability of intermediate hosts, primarily soil-dwelling oligochaetes such as earthworms [32], whose abundance is influenced by soil characteristics, humidity, and climate. Host foraging behavior and seasonal environmental variation probably further shape infection dynamics. Notably, despite its moderate prevalence, *C. contorta* was implicated in 42.02% of parasite-associated mortalities within co-infections, revealing a marked discrepancy between ecological frequency and pathological impact. This observation is consistent with previous reports identifying *C. contorta* among the most pathogenic nematodes of Galliformes [31,33,34,35]. However, the degree of contribution of *C. contorta* to mortality cannot be isolated from the synergistic effects of co-infecting parasites.

A key finding of this study is the consistent occurrence of *C. contorta* within mixed infections; no mono-infections were detected, and prevalence increased with infection complexity. Frequent associations with *S. trachea* and *Eimeria* spp. likely contributed to enhanced disease severity. Similar patterns have been reported in other *Alectoris* species, which commonly harbor diverse parasite assemblages [14,21]. Co-infections involving *Capillaria* spp. have also been reported in galliform birds by several authors [35,36,37]. Máca and Pavlásek [12], studying aviary-reared *Alectoris rufa*, documented persistent mixed infections involving *Capillaria* spp., *S. trachea*, and *Eimeria* spp., except during antiparasitic treatment. These findings closely parallel ours and underscore the epidemiological and pathological importance of polyparasitism. Earlier work at the same study site [21] also reported co-infections between *Capillaria* and *Eimeria* spp., suggesting long-term stability of this parasite community within the semi-captive system. It would also be of great interest to study the wild populations of *A. barbara*, as well as the populations released from breeding centers. These wild populations are subject to other ecological factors that could alter the composition of their helminth faunas.

Temporal analysis demonstrated year-round persistence of *C. contorta* over the four-year study period, although a significant decline in prevalence was observed from 2021 to 2024. Given the large sample size and narrowing confidence intervals in later years, this reduction likely reflects a genuine epidemiological trend rather than sampling bias. Potential explanations might include improved sanitary measures or modifications in management practices. Interannual climatic variability may also have influenced egg survival and embryonation, as *C. contorta* eggs are sensitive to fluctuations in temperature and humidity [19].

Seasonally, infections peaked in spring and declined during summer and autumn. This pattern is consistent with environmental modulation of the parasite’s life cycle and intermediate host activity [7,38]. According to Svoboda in 1992 [39], higher temperatures and rainfall favor the appearance of various parasites. Moderate temperatures and increased humidity during spring in the Algiers Sahel region likely favor egg survival and earthworm abundance, enhancing transmission. Comparable seasonal trends have been reported in poultry from Tunisia [40], which shares similar Mediterranean climatic conditions. In contrast, studies from tropical regions have documented different seasonal peaks, such as autumn in Kashmir [41] and summer in southern India [42], highlighting the strong influence of regional climate on *Capillaria* epidemiology. Similarly, in Cantabrian capercaillie (*Tetrao urogallus*), higher prevalence at lower altitudes has been linked to humidity- and temperature-dependent variation in intermediate host availability [19].

The histopathological lesions observed were consistent with previous descriptions in other avian hosts infected with *C. contorta*. Adult worms embedded within epithelial tunnels, accompanied by eggs dispersed in hyperplastic stratified squamous epithelium, have been reported in teal ducks (*Anas crecca*) [43] and red-legged partridges (*A. rufa*) [11]. Parasite measurements in our study fall within previously reported ranges [44,45,46,47], supporting accurate taxonomic identification. Unlike some reports, no involvement of the proventriculus was observed in our samples, suggesting possible host-specific differences or environmental influences on parasite localization. Notably, *C. contorta* has been reported to affect the proventriculus in other galliform species. For example, [11] described outbreaks of capillariasis in farm-raised red-legged partridges (*A. rufa*) in Spain. In that study, parasites and eggs were consistently detected in the epithelium of the esophagus and crop, with adult worms located in the basal epithelial layers and eggs distributed in the intermediate layers. Additionally, *C. contorta* was observed in the anterior region of the proventriculus, where it induced epidermoid metaplasia, a lesion distinct from those observed in the esophagus and crop, which were characterized primarily by epithelial spongiosis. The absence of proventricular involvement in the present study may reflect potential differences in host species, infection intensity, environmental conditions, or management practices influencing parasite distribution within the digestive tract.

The persistence of *C. contorta* over more than a decade at the study site, as indicated by earlier preliminary surveys [21], suggests stable establishment within the semi-captive system. Chronic inflammatory lesions, including lymphoplasmacytic infiltration, epithelial hyperplasia, and submucosal fibrosis, indicate prolonged and repeated exposure. These pathological features reflect cumulative tissue damage and are consistent with chronic capillariosis described in other galliform hosts [43]. Congestion and hemorrhage both prevent normal feeding function, compromising nutritional status and contributing to mortality. This direct correlation suggests that *C. contorta* is a significant pathogenic agent capable of causing mortality. Finally, management practices appear central to maintaining transmission. While reproductive stress and rearing conditions may predispose birds to infection [48], soil access likely represents the primary determinant of persistence by facilitating continuous exposure to infective eggs and intermediate hosts. Soil may potentially function as a long-term reservoir, which could enable transmission between successive cohorts. Experimental evidence supports this interpretation: Cruz et al. [48] demonstrated that transferring *E. contortus* (syn. *C. contorta*)-infected quail to raised, wire-floor systems interrupted transmission and prevented mortality. These findings emphasize the importance of management strategies that limit soil contact to control capillariosis in semi-captive galliform populations. It will be of interest to explore the helminth fauna of the Barbary partridge in wild populations, where some of the factors that are found in semi-captive populations can differ from previous studies in farms (for example, in Tenerife Island [22]), as a result of changes in ecological factors, including reduced host density in the wild and limited availability of diverse food resources.

### Limitations of the Study

Morphological and histopathological findings support identification consistent with *C. contorta*. Although *C. contorta* and *C. annulata* share the same anatomical predilection sites (crop and esophagus), they differ in the presence of a cephalic cuticular swelling, which is absent in *C. contorta* [7,49]. While this morphological feature remains important for classical diagnosis, molecular approaches are increasingly recommended due to the high morphological similarity among capillariid species [50,51]. Our identification is based exclusively on morphological and morphometric criteria. This limitation presents an important avenue for future research, particularly through the use of molecular markers such as DNA sequencing and 18S rRNA to confirm the identification and eliminate any risk of interspecific confusion, especially in cases in which the materials for morphological examination are scarce. Our repeated sampling from the same aviaries, combined with the pooling of fecal samples, results in non-independent observations within each aviary over time. This pseudo-replication breaches the independence assumptions required for robust statistical tests, such as multivariate approaches. While chi-square tests comparing prevalence between seasons and years represent the most appropriate approach available for our pooled aviary-level data, this sampling design limitation should be considered when interpreting statistical comparisons.

## 5. Conclusions

This study presents the first integrated eco-epidemiological and histopathological investigation of *C. contorta* infection in the Barbary partridge (*A. barbara*) in Algeria, providing new insights into the dynamics and pathological significance of this parasite in a semi-captive system. Although classified as a satellite species based on its prevalence, *C. contorta* exhibited marked pathological importance, contributing substantially to parasite-associated mortality and demonstrating a clear imbalance between epidemiological frequency and clinical impact. The persistent detection of the parasite over four consecutive years, together with pronounced seasonal variation characterized by spring peaks and its systematic occurrence in mixed infections, highlights the complexity of transmission dynamics in semi-captive environments. These findings emphasize the role of environmental factors, host behavior, and co-infection processes in shaping parasite persistence and disease expression.

Histopathological evidence of severe chronic lesions affecting the crop and esophagus confirms the significant clinical impact of capillariosis and underscores the consequences of prolonged and repeated exposure. The observed decline in prevalence over time further suggests that improvements in management practices, sanitary measures, and possibly targeted treatments can effectively reduce infection pressure and mitigate disease outcomes.

Overall, these results stress the importance of implementing integrated surveillance and control strategies combining parasitological monitoring, environmental management, and optimized husbandry practices. Such approaches are essential not only to safeguard animal health and welfare and improve productivity in breeding programs but also to minimize the risk of parasite transmission to wild populations, particularly in the context of restocking and conservation initiatives. Future studies incorporating wild and restocking birds, as well as the search for intermediate hosts that participate in the parasite cycles, would further refine our understanding of *C. contorta* epidemiology and other helminths and support the development of more effective control strategies.

## Figures and Tables

**Table 1:** Annual aviary-level prevalence of *Capillaria contorta* infection based on coprological examination in semi-captive *A. barbara* from Zeralda Hunting Center in Algeria (2021–2024). Total positive: total number of samples positive for at least one parasite per year. * *p*-value < 0.05.

Year	*C. contorta* Positive (*n* = 278)	Total Positive (*n* = 844)	Prevalence (%)	95 CI	χ^2^	*p*-Value
2021	37	94	39.36%	[29.49–49.24%]	18.820	0.000297 *
2022	130	342	38.01%	[32.87–43.16%]		
2023	80	249	32.13%	[26.33–37.93%]		
2024	31	159	19.50%	[13.34–25.65%]		

**Table 2:** Seasonal variation in aviary-level *Capillaria contorta* prevalence based on coprological examination in semi-captive *A. barbara* from Zeralda Hunting Center in Algeria (2021–2024). Total positive: total number of samples positive for at least one parasite per season. * *p*-value < 0.05.

Season	*C. contorta* Positive (*n* = 278)	Total Number of Positive (*n* = 844)	Prevalence (%)	95 CI	χ^2^	*p*-Value
Autumn	25	140	17.86%	[11.51–24.2%]	93.189	0.000000 *
Summer	20	151	13.25%	[7.84–18.65%]		
Winter	57	206	27.67%	[21.56–33.78%]		
Spring	176	347	50.72%	[45.46–55.98%]		

## Data Availability

Data are presented in the manuscript.

## Appendix A

**Table A1:** Co-infection patterns of *Capillaria contorta* with other parasitic taxa based on coprological examination (% prevalence). *Cap*., *Capillaria*; *Strong* sp., *Strongyloides* sp.; *Tricho te.*, *Trichostrongylus tenuis*; *Amido an.*, *Amidostomum anseris; Asca ga.*, *Ascaridia galli*; *Syng tr.*, *Syngamus trachea*; *Het gal.*, *Heterakis gallinarum*; *Tric* sp., *Trichuris* sp. (spurious parasitism); *Hyme* sp., *Hymenolepis* sp.; *Eimeria* spp. (*E. lyruri*, *E. kofoidi*, *Eimeria* spp.); *Aca* spp., *Acarina* spp.

Capillaria + Species Number	Prevalence (*p* %)	Species Composition	*n* Infected Prevalence	*p* %
*Capillaria*	0.00	*Cap.*	0	0.00
Mixed *Capillaria* + 1 species	2.52	*Syng tr.*	2	0.72
		*Eimeria* spp.	3	1.08
		*Aca* spp.	2	0.72
Mixed *Capillaria* + 2 species	8.99	*Strong sp.*-*Aca* spp.	1	0.36
		*Syng tr.*-*Tricho te.*	4	1.44
		*Syng tr.*-*Amido an.*	1	0.36
		*Syng tr.*-*Eimeria* spp.	2	0.36
		*Syng tr.*-*Acar* spp.	1	1.08
		*Syng tr.*-*Het* spp.	1	0.72
		*Amido an.*-*Het gal.*	1	0.72
		*Amido an.*-*Aca* spp.	3	0.36
		*Amido an.*-*Tric* sp.	2	0.36
		*Eimeria* spp.-*Hyme* sp.	1	0.36
		*Asca ga.*-*Tric* sp.	2	0.72
		*Eimeria* spp.-*Aca* spp.	6	2.16
Mixed *Capillaria* + 3 species	14.03	*Strong* sp.-*Tricho te.*-*Amido an.*	1	0.36
		*Strong* sp.-*Tricho te.*-*Syng tr.*	1	0.36
		*Strong* sp.-*Tricho te.*-*Het gal.*	2	0.72
		*Strong* sp.-*Amido an.*-*Syng tr.*	10	3.60
		*Strong* sp.-*Amido an.*-*Eimeria* spp.	1	0.36
		*Strong* sp.-*Het gal.*-*Eimeria* spp.	1	0.36
		*Strong* sp.-*Eimeria* spp.-*Aca* spp.	5	1.80
		*Strong* sp.-*Tric* sp.-*Eimeria* spp.	5	1.80
		*Strong* sp.-*Hyme* sp.-*Eimeria* spp.	2	0.72
		*Tricho te.*-*Het gal.*-*Tric* sp.	2	0.72
		*Tricho te.*-*Syng tr.*-*Eimeria* spp.	1	0.36
		*Tricho te.*-*Amido an.*-*Eimeria* spp.	2	0.72
		*Amido an.*-*Eimeria* spp.-*Aca* spp.	3	1.08
		*Syng tr.*-*Het gal.*-*Tric* sp.	1	0.36
		*Syng tr.*-*Eimeria* spp.-*Aca* spp.	1	0.36
		*Tric* sp.-*Hyme* sp.-*Eimeria* spp.	1	0.36
Mixed *Capillaria* + 4 species	19.06	*Strong* sp.-*Tricho te.*-*Amido an.*-*Syng tr.*	4	1.44
		*Strong* sp.-*Tricho te.*-*Amido an.*-*Eimeria* spp.	4	1.44
		*Strong* sp.-*Tricho te.*-*Syng tr.*-*Het gal.*	4	1.44
		*Strong* sp.-*Tricho te.*-*Syng tr.*-*Eimeria* spp.	3	1.08
		*Strong* sp.-*Tricho te.*-*Syng tr.*-*Aca* spp.	1	0.36
		*Strong* sp.-*Tricho te.*-*Hyme* sp.-*Eimeria* spp.	2	0.72
		*Strong* sp.-*Asca ga.*-*Tric* sp.-*Eimeria* spp.	2	0.72
		*Strong* sp.-*Amido an.*-*Asca ga.*-*Tric* sp.	1	0.36
		*Strong* sp.-*Asca ga.*-*Syng tr.*-*Eimeria* spp.	2	0.72
		*Strong* sp.-*Syng tr.*-*Tric* sp.-*Aca* spp.	1	0.36
		*Strong* sp.-*Syng tr.*-*Het gal.*-*Tric* sp.	1	0.36
		*Strong* sp.-*Het gal.*-*Tric* sp.-*Aca* spp.	1	0.36
		*Strong* sp.-*Tric* sp.-*Hyme* sp.-*Eimeria* spp.	1	0.36
		*Strong* sp.-*Tric* sp.-*Eimeria* spp.-*Aca* spp.	1	0.36
		*Strong* sp.-*Syng tr.*-*Eimeria* spp.-*Aca* spp.	1	0.36
		*Strong* sp.-*Syng tr.*-*Het gal.*-*Aca* spp.	1	0.36
		*Strong* sp.-*Amido an.*-*Syng tr.*-*Het gal.*	1	0.36
		*Strong* sp.-*Amido an.*-*Asca ga.*-*Het gal.*	2	0.72
		*Tricho te.*-*Amido an.*-*Eimeria* spp.-*Aca* spp.	1	0.36
		*Tricho te.*-*Asca ga.*-*Syng tr.*-*Eimeria* spp.	2	0.72
		*Tricho te.*-*Asca ga.*-*Eimeria* spp.-*Aca* spp.	1	0.36
		*Tricho te.*-*Syng tr.*-*Eimeria* spp.-*Aca* spp.	6	2.16
		*Tricho te.*-*Tric* sp.-*Eimeria* spp.-*Aca* spp.	3	1.08
		*Asca ga.*-*Syng tr.*-*Het gal.*-*Eimeria* spp.	2	0.72
		*Asca ga.*-*Syng tr.*-*Tric* sp.-*Aca* spp.	1	0.36
		*Syng tr.*-*Tric* sp.-*Eimeria* spp.-*Aca* spp.	3	1.08
		*Amido an.*-*Syng tr.*-*Het gal.*-*Tric* sp.	1	0.36
